# Supplementary material for: Rich spatio-temporal stimulus dynamics unveil sensory specialization in cortical area S2
Source: Nat Commun. 2018 Oct 3;9:4053. doi: 10.1038/s41467-018-06585-4 (PMC6170455; doi:10.1038/s41467-018-06585-4)
Supplement: Supplementary file 1 — Supplementary Information [file 41467_2018_6585_MOESM1_ESM.pdf]

Article title:

Rich spatio-temporal stimulus dynamics unveil sensory specialization in cortical area S2

Author names:

Matías A. Goldin[†], Evan R. Harrell[†], Luc Estebanez, Daniel E. Shulz*

[†]these authors contributed equally

Author affiliation:

Unité de Neurosciences, Information et Complexité, UNIC-FRE3693, Centre National de la Recherche Scientifique, Gif sur Yvette, 91198, France

Corresponding author:

*shulz@unic.cnrs-gif.fr

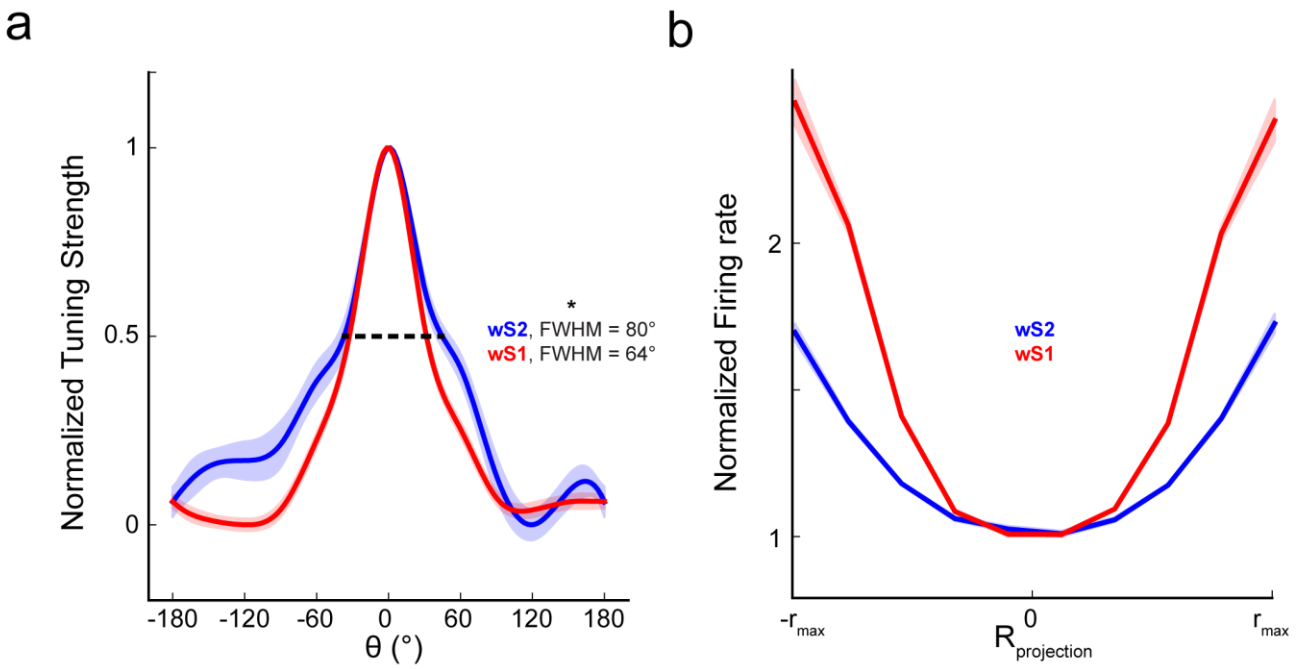

Supplementary Figure 1. Non-linear functions (**a**) Average phase tuning for phase-selective cells in wS2 (blue, n=26) and wS1 (red, n=167). Dark lines are the mean and shaded regions are standard errors, cells were aligned by their preferred frequency. The full-width half maximum is larger in wS2 by 16° (p_{val} = 0.018, permutation test). (**b**) Population average 1-D non-linear functions for the phase-invariant cells in wS2 (blue, n=173) and wS1 (red, n=365). The full 2-D non-linear functions (as shown in Fig. 2) were collapsed by averaging across phase complements. Dark lines are mean and shaded areas are standard errors. Firing rates were also normalized to the baseline firing rate of each cell. The wS1 cells fire more for a filter-like stimulus than wS2.

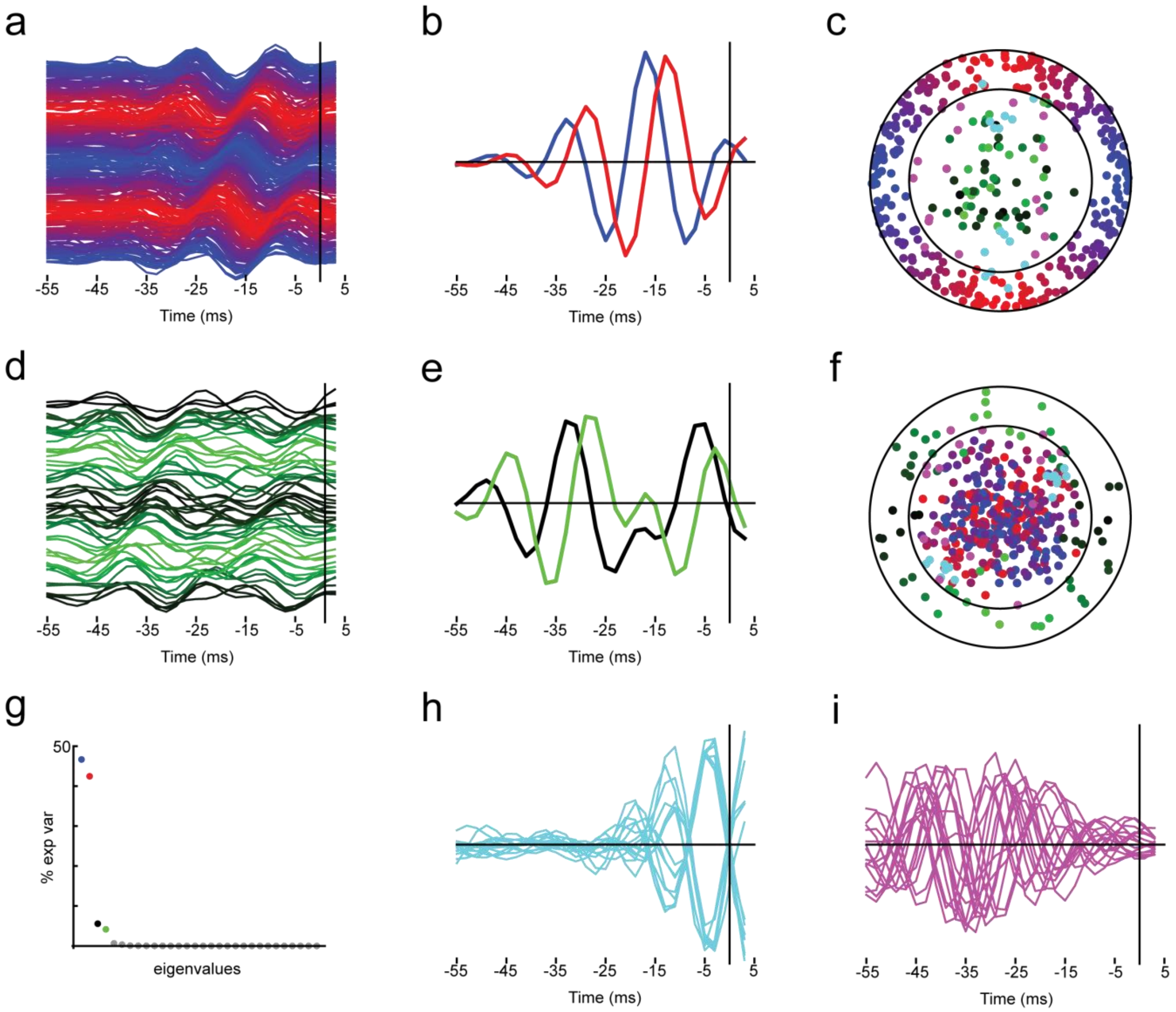

Supplementary Figure 2. Significant filters: relevant, complementary, fast and slow in wS2. All the filters in this figure have been obtained with a 55ms pre- and 5ms post-spike window to capture the full behavior of wS2 filters. (**a**) All 328 filters offset and colored by phase from the relevant subspace of wS2 (from 189 neurons). (**b**) Relevant subspace population filters: First two principal components of PCA made on all wS2 filters. (**c**) Projected filters into the relevant subspace **b** of wS2. All filters well-represented in this subspace fall near the unit circle. Colors are as in **a**, plus green, black, cyan and magenta which represent filters that are less well-represented. Inner circle is for reference, at a 0.7 radius value. (**d**) All 57 complementary filters offset and colored by phase from the complementary subspace of wS2 (from 38 neurons). (**e**) Third and fourth principal components of PCA made on all wS2 filters, same colors as in **c-d**. (**f**) Projected filters into the additional subspace **e** of wS2. All filters well represented in this subspace fall near the unit circle, same colors as in **c**. (**g**) First two PCA filters represent about 90% of the variance of all filters, while third and fourth explain the remaining 10%. (**h**) All 15 fast filters that do not fall in the relevant or the complementary subspace of wS2, same colors as in **c** and **f** (15 neurons). (**i**) All 11 slow filters that do not fall in the relevant nor the complementary subspace of wS2, same colors as in **c** and **f**. (7 neurons).

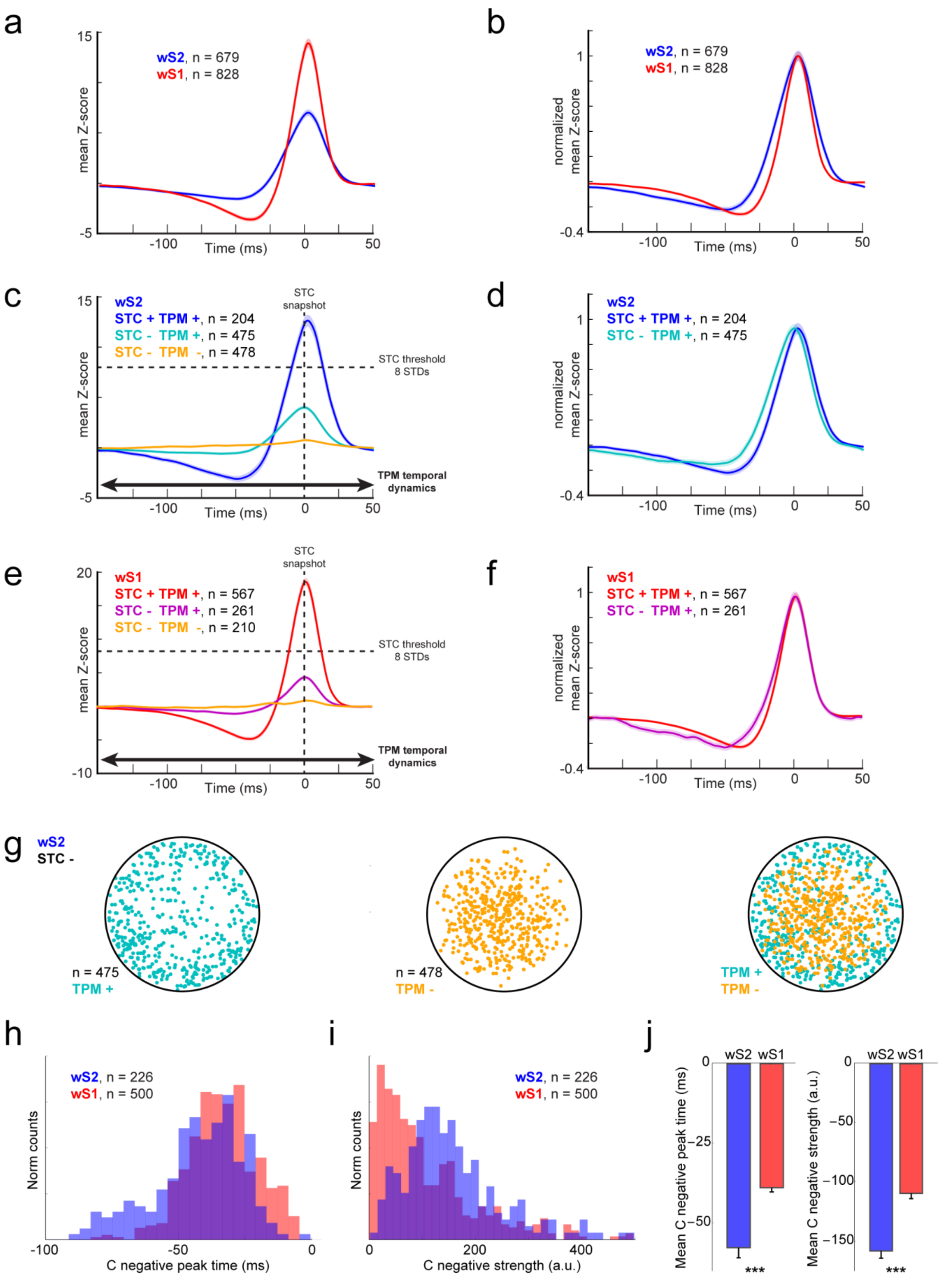

Supplementary Figure 3. PS-STA population averages, tail area and negative peak time in correlated stimulation. (**a**) Population average PS-STAs for all wS2 (blue, n=679) and wS1 (red, n=828) cells giving significant responses. Dark lines are means and shaded regions are standard errors. (**b**) Population average PS-STAs normalized to the maximum

for easier comparison for all wS2 (blue, n=679) and wS1 (red, n=828) cells giving significant responses. Dark lines are means and shaded regions are standard errors. (**c**) PS-STA population averages in wS2 for cells that gave significant filters from spike-triggered covariance analysis (blue curve, mean firing rate 2.9 Hz), cells that had significant PS-STAs but did not give STC filters (cyan curve, mean firing rate 1.6 Hz), and cells that had no significant response to either analysis method (orange curve, mean firing rate 0.8 Hz). The STC threshold of 8 standard deviations above the shuffled distribution is shown on the graph to illustrate that cells that do not have significant STC filters have weaker PS-STA responses, but the responses are present. STC takes a snapshot only at time=0 on this graph and the TPM looks at the full temporal dynamics. (**d**) The same as **c** but normalized to the maximum to highlight shape differences between the PS-STAs of wS2 cells that give STC filters and those that do not give STC filters. (**e-f**) The same as **c-d** but for wS1 (mean firing rates are 2.0, 1.1, and 0.6 Hz for red, magenta and orange respectively). (**g**) Left: The eigenvector with the largest eigenvalue from cells with significant PS-STAs that did not give STC filters (cyan) in wS2 projected into the relevant filter subspace shown in Figure 1. Center: The same as the left but for cells that did not have significant PS-STAs or give STC filters. Right: Superposition of the two graphs to the left. **(h-i)** Distribution of the negative peak time (**h**) and the negative integrated area of the PS-STA curve (**i**) for the correlated stimulation for wS2 (blue) and wS1 (red). (**j**) Mean population values present significant differences for the negative peak time (left) and the negative strength (right) between wS2 and wS1 (Mann–Whitney–Wilcoxon two-sample rank sum test, p=3e-16 and p=1e-13). Error bars are standard errors. These measures were not selected as general features (see Supplementary Fig. 4) because not all neurons exhibited them: one fifth and almost half of the cells for wS2 and wS1 respectively.

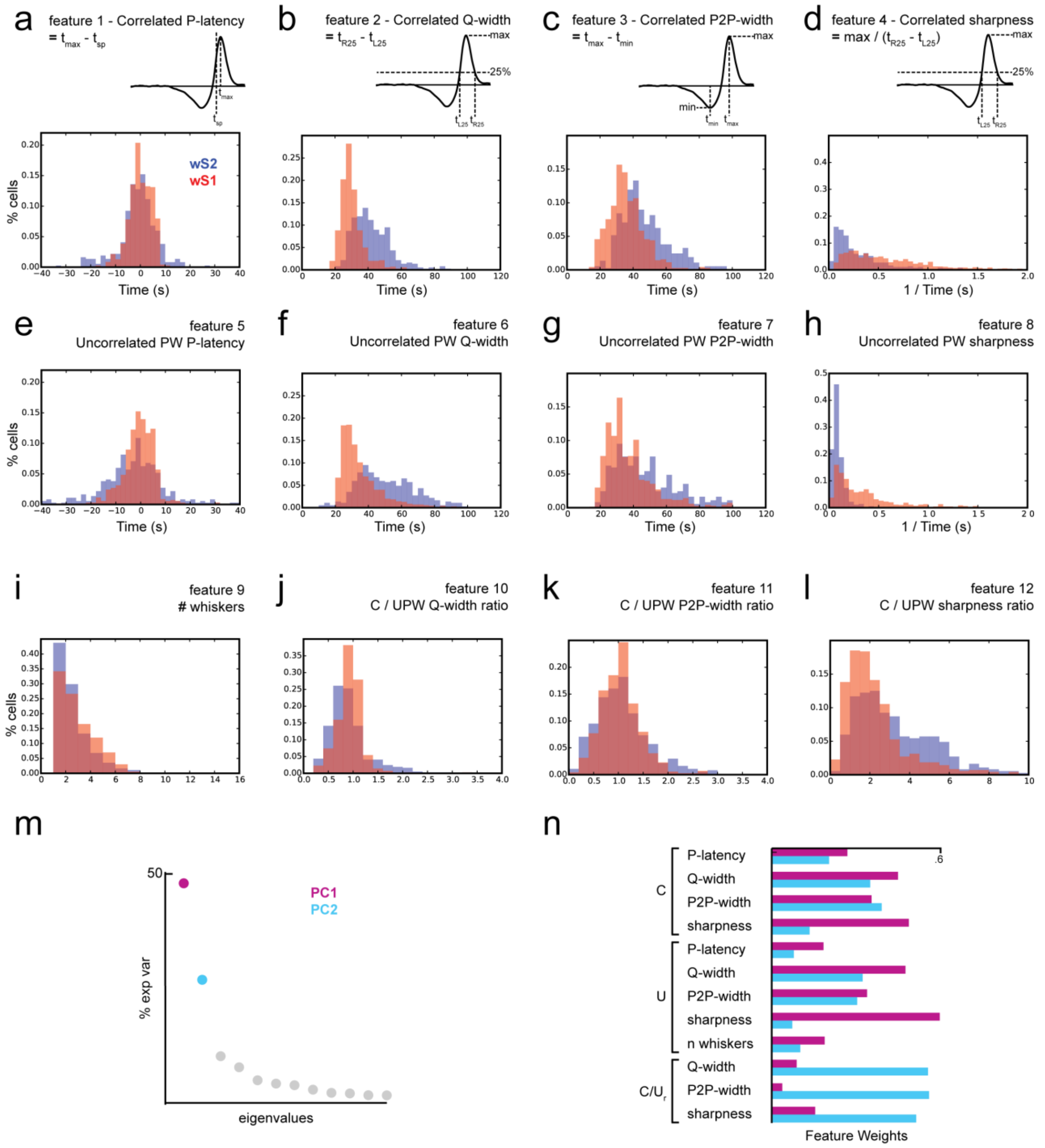

Supplementary Figure 4. **PS-STA response features** (**a**) Top: Correlated P-latency: peak latency is the time difference between the spike (t_{sp}) time and peak of the PS-STA profile (t_{max}) in the correlated stimulation. Bottom: Histograms showing wS2 (blue) and wS1 (red) populations. These are not corrected for time differences in relevant filters, so they are different from Fig. 4. (**b**) Top: Correlated Q-width: quarter-width is the width of the PS-STA profile at 25% of the maximum peak. Bottom: Histograms showing wS2 (blue) and wS1 (red) populations. (**c**) Top: Correlated P2P-width: peak to peak width is the distance between the positive peak (t_{max}) and the negative peak (t_{min}) in the PS-STA profile. Bottom: Histograms showing wS2 (blue) and wS1 (red) populations. (**d**) Top: Correlated sharpness: sharpness is the ratio between the height of the positive peak of the PS-STA and the Q-width. Bottom: Histograms showing wS2 (blue) and wS1 (red) populations. (**e-h**) Features 5-8 are the same as features 1-4, but they are applied to the PS-STA profile of the principal whisker (PW) in the uncorrelated stimulation. All histograms show wS2 (blue) and wS1 (red) populations. (**i**) Feature 9 is the number of significant whiskers found during

uncorrelated stimulation. (**j-l**) Features 10 to 12 are the ratios between features 2-4 and 6-8 respectively that quantify differences between the correlated and uncorrelated stimulation. (**m**) The eigenvalues obtained from a PCA on all features. The first two principal components contain ~80% of the variance. They are used for the presentation in Fig. 5. (**n**) The absolute weights for the first two principal components.

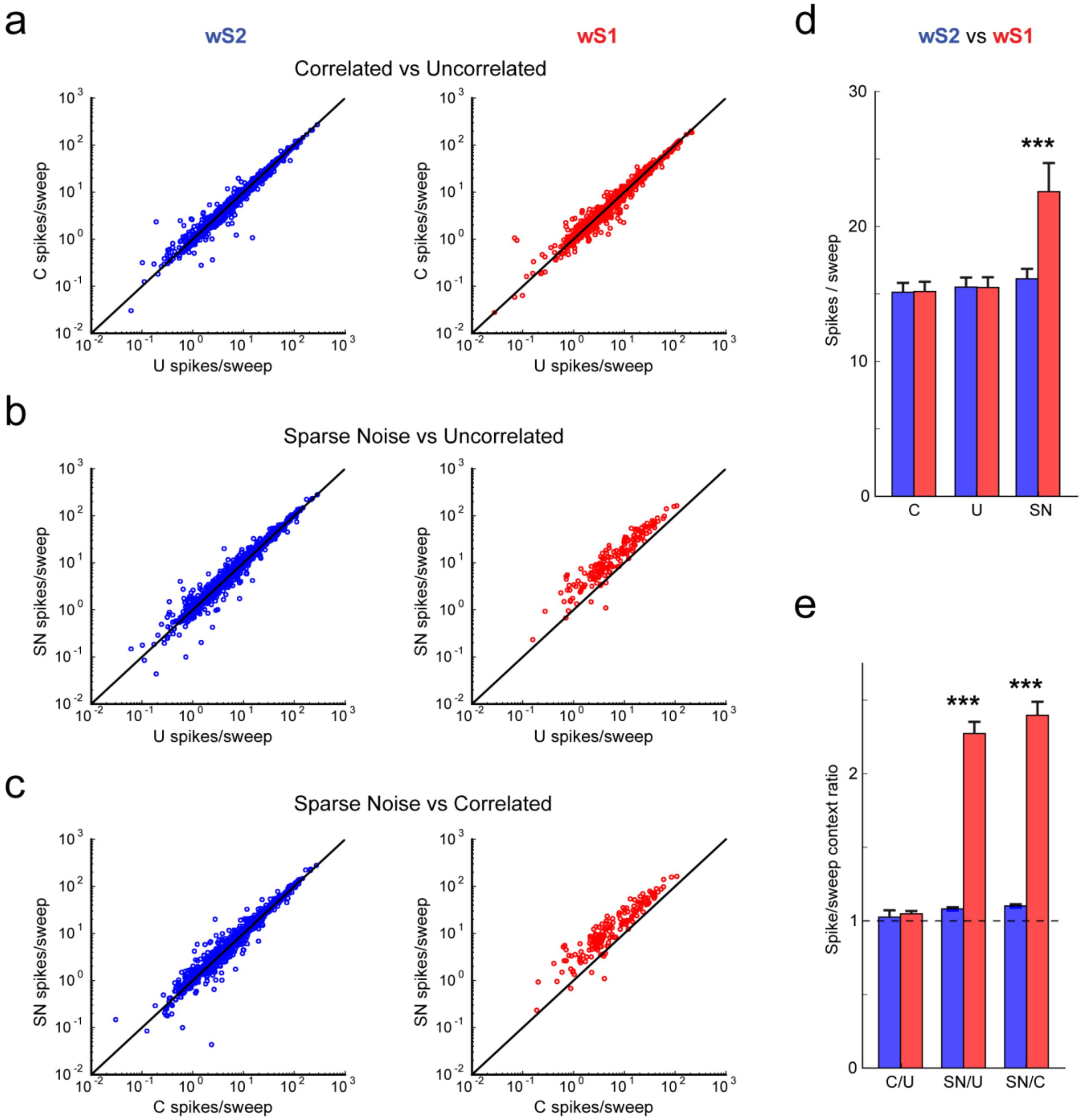

Supplementary Figure 5. Firing rate comparisons among the three different stimulation patterns for all neurons. (a) Firing rate relationships between correlated (C) and uncorrelated stimulation (U) for wS2 (left) and wS1 (right). Cells from both cortical areas align well around the identity line. **(b)** Firing rate relationships between sparse noise stimulation (SN) and uncorrelated stimulation (U) for wS2 (left) and wS1 (right). wS1 presents a higher firing rate in the sparse noise stimulation and wS2 does not. **(c)** Firing rate relationships between sparse noise stimulation (SN) and correlated stimulation (C) for wS2 (left) and wS1 (right). wS1 presents a higher firing rate in the sparse noise stimulation and wS2 does not. (**d**) Quantification of the spikes per repetition mean response of the cells in the three patterns of stimulation (wS2 in blue, wS1 in red). The only stimulation pattern and cortical area presenting significant differences from the others was wS1 in the sparse noise stimulation ($p < 0.001$, two tailed t-test). Error bars are standard errors. (**e**) Mean ratio between firing in different stimulation patterns (wS2 in blue, wS2 in red). Significant increase in firing rate was only found for wS1 in the sparse noise stimulation, which more than doubled the firing rate compared to the correlated or the uncorrelated stimulation ($p<0.001$ two tailed t-test). Error bars are standard errors.

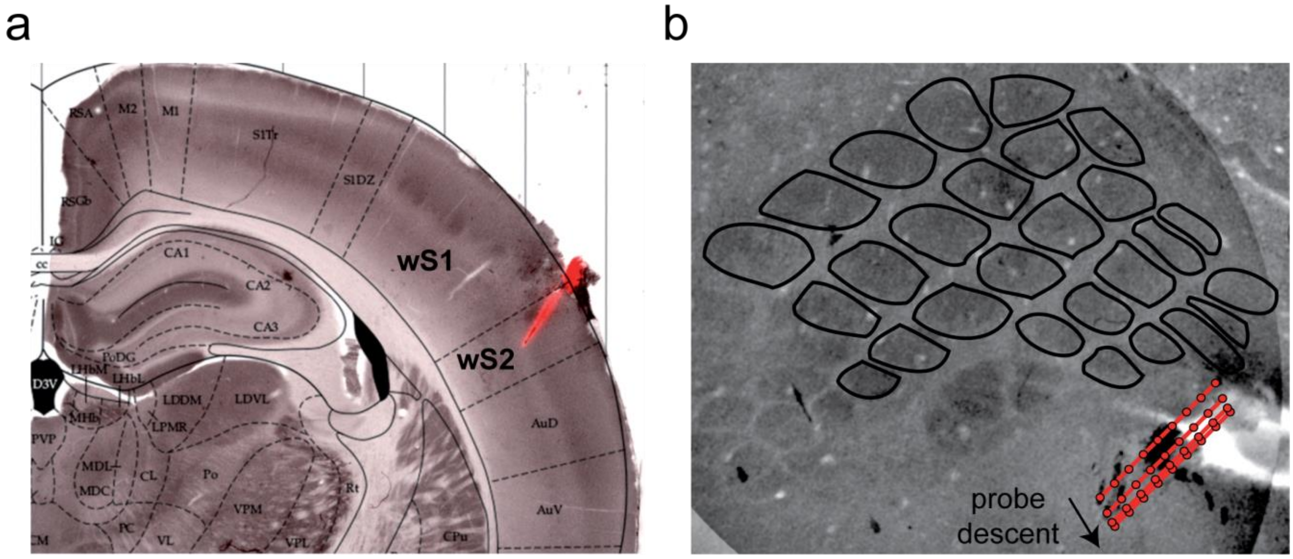

Supplementary Figure 6. Electrode recording sites identified histologically. Cytochrome oxidase stained barrel cortex structures and DiI fluorescent traces in red made by the electrode shanks in: (**a**) A coronal cut showing a single electrode penetration. (**b**) A merged image of four different slices from a flattened cortex. Red circles represent the position of the eight shanks in each slice, which were joined with a red line for viewing assistance. The barrels were delineated for the same purpose. Units recorded in the experiment where **a** was obtained had responses only from D and E row stimulation. Right-most four electrodes in the experiment where **b** was obtained showed no responses, confirming that recorded signals only came from wS2.

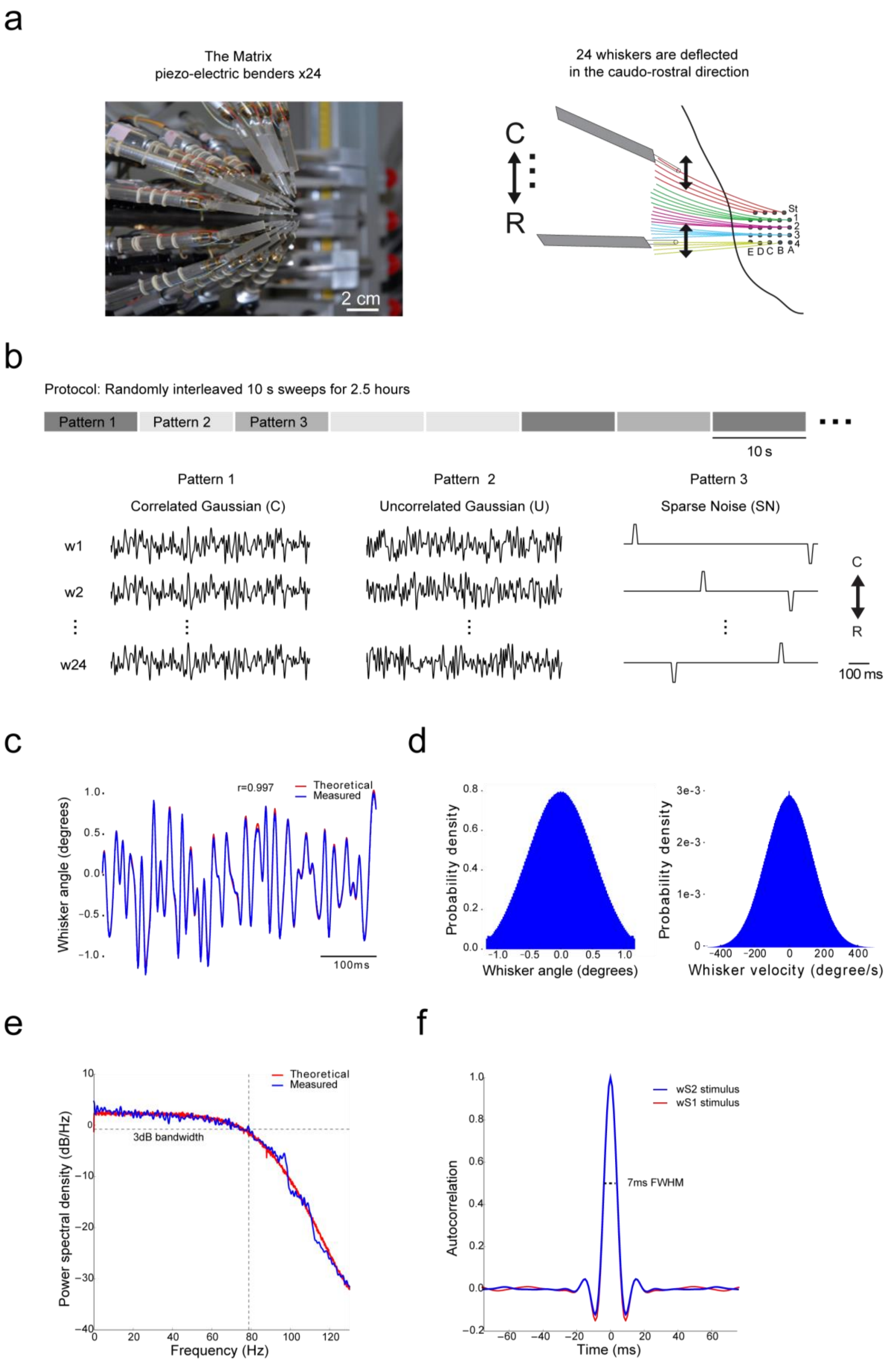

Supplementary Figure 7. Stimulus characterization. (**a**) Top left: Frontal picture of matrix of piezo-electric stimulator benders (from Jacob et al. 2010). Top right: Schematic top view of a row of piezo-electric benders with whiskers inserted into their tips. Whiskers are cut to 10 mm length and inserted 3 mm (held 7 mm from follicle). The whisker pad

arrangement corresponds to the matrix elements (St: straddlers, 1-4 Arcs, A-E rows). Motion is in the rostro-caudal direction, from each whisker resting position. (**b**) Top: Schematic of the protocol, where different stimulation patterns are interleaved during 2.5 hrs. Each repetition has 10 s duration. Bottom: Example matrix movement commands for Gaussian white noise for correlated (left) and uncorrelated (middle) stimulation. Each trace is a Gaussian white noise stimulus, identical for every whisker in correlated stimulation and different in uncorrelated stimulation. Sparse noise (right) is a succession of individual whisker movements spaced by 50ms and elicited randomly. Stimulus profile is a 10-10-10 ms ramp-hold-ramp. (**c**) Measured piezo-electric movement at the tip overlaid with the matrix theoretical command. (**d**) Distribution of whisker positions in angular coordinates for the Gaussian white noise stimulus (left) and the velocities sampled (right). (**e**) Spectral density characteristics of both theoretical and measured piezo-electric movements. A 3dB bandwidth was measured up to 79 Hz. (**f**) Autocorrelation for both wS2 and wS1 Gaussian white noise stimulus show an FWHM value of 7 ms, meaning that any correlations extending outside of this region are not stimulus artifacts.
