# Supplementary material for: Rich spatio-temporal stimulus dynamics unveil sensory specialization in cortical area S2
Source: Nat Commun. 2018 Oct 3;9:4053. doi: 10.1038/s41467-018-06585-4 (PMC6170455; doi:10.1038/s41467-018-06585-4)
Supplement: Supplementary file 3 — Description of Additional Supplementary Files [file 41467_2018_6585_MOESM3_ESM.pdf]

SUPPLEMENTARY VIDEO LEGENDS

Supplementary Movie 1. Dynamic receptive field for the wS2 regular spiking neuron from Figures 2,3,4,5 and 6.

Supplementary Movie 2. Dynamic receptive field for the wS1 regular spiking neuron from Figures 2,3,4,5 and 6.

Supplementary Movie 3. Dynamic receptive field for a unique wS2-like regular spiking neuron.

Supplementary Movie 4. Dynamic receptive field for a unique wS1-like regular spiking neuron.

Supplementary Movie 5. Dynamic receptive field for a regular spiking wS2 neuron where a moving pattern of high PS-STA values can be observed across the receptive field.

Supplementary Movie 6. Dynamic receptive field for a regular spiking wS1 neuron.

Supplementary Movie 7. Dynamic receptive field for a unique wS2-like fast spiking neuron.

Supplementary Movie 8. Dynamic receptive field for a unique wS1-like fast spiking neuron lacking a negative tail.

Supplementary Movie 9. Dynamic receptive field for a fast spiking wS2 neuron showing a large receptive field.

Supplementary Movie 10. Dynamic receptive field for a fast spiking wS1 neuron showing a large receptive field.
